# Two Routes to Genetic Suppression of RNA Trimethylguanosine Cap Deficiency via C-Terminal Truncation of U1 snRNP Subunit Snp1 or Overexpression of RNA Polymerase Subunit Rpo26

**DOI:** 10.1534/g3.115.016675

**Published:** 2015-04-24

**Authors:** Zhicheng R. Qiu, Beate Schwer, Stewart Shuman

**Affiliations:** * Molecular Biology Program, Sloan-Kettering Institute, New York, New York 10065; † Department of Microbiology and Immunology, Weill Cornell Medical College, New York, New York 10065

**Keywords:** trimethylguanosine synthase, U1 snRNP, RNA polymerase subunit, Rpo26

## Abstract

The trimethylguanosine (TMG) caps of small nuclear (sn) RNAs are synthesized by the enzyme Tgs1 via sequential methyl additions to the N2 atom of the m^7^G cap. Whereas TMG caps are inessential for *Saccharomyces cerevisiae* vegetative growth at 25° to 37°, *tgs1*∆ cells that lack TMG caps fail to thrive at 18°. The cold-sensitive defect correlates with ectopic stoichiometric association of nuclear cap-binding complex (CBC) with the residual m^7^G cap of the U1 snRNA and is suppressed fully by Cbc2 mutations that weaken cap binding. Here, we show that normal growth of *tgs1*∆ cells at 18° is also restored by a C-terminal deletion of 77 amino acids from the Snp1 subunit of yeast U1 snRNP. These results underscore the U1 snRNP as a focal point for TMG cap function *in vivo*. Casting a broader net, we conducted a dosage suppressor screen for genes that allowed survival of *tgs1*∆ cells at 18°. We thereby recovered *RPO26* (encoding a shared subunit of all three nuclear RNA polymerases) and *RPO31* (encoding the largest subunit of RNA polymerase III) as moderate and weak suppressors of *tgs1*∆ cold sensitivity, respectively. A structure-guided mutagenesis of Rpo26, using *rpo26*∆ complementation and *tgs1*∆ suppression as activity readouts, defined Rpo26-(78-155) as a minimized functional domain. Alanine scanning identified Glu89, Glu124, Arg135, and Arg136 as essential for *rpo26*∆ complementation. The *E124A* and *R135A* alleles retained *tgs1*∆ suppressor activity, thereby establishing a separation-of-function. These results illuminate the structure activity profile of an essential RNA polymerase component.

Trimethylguanosine (TMG) cap structures are characteristic of small nuclear (sn) RNAs, small nucleolar (sno) RNAs, and telomerase RNA. TMG is formed post-transcriptionally by the enzyme Tgs1, which catalyzes two successive methyl additions to the N2 atom of the m^7^G cap (Mouaikel *et al.* 2002; Hausmann and Shuman 2005; Chang *et al.* 2010). Whereas m^7^G caps are essential for the viability of eukaryal cells, TMG caps are not (Mouaikel *et al.* 2002; Hausmann *et al.* 2007). *Saccharomyces cerevisiae* and *Schizosaccharomyces pombe tgs1*∆ cells have no detectable TMG caps on their snRNAs, snoRNAs, or telomerase RNA, signifying that there is no Tgs1-independent route to form TMG caps (Mouaikel *et al.* 2002; Hausmann *et al.* 2007; Gallardo *et al.* 2008; Franke *et al.* 2008; Simoes-Barbosa *et al.* 2012). *tgs1*∆ yeast cells display apparently normal steady-state snRNA levels (Mouaikel *et al.* 2002; Hausmann *et al.* 2007) and no overt aberrations in the RNA or protein contents of their spliceosomal snRNPs, except for the acquisition of the nuclear cap-binding complex (CBC) as a stoichiometric component of the U1 snRNP (Schwer *et al.* 2011).

*S. cerevisiae* can grow in the absence of Tgs1 because the effects of ablating the TMG cap of the spliceosomal U snRNAs are genetically buffered, either by spliceosome assembly factors that are themselves inessential for vegetative growth (Hausmann *et al.* 2008; Wilmes *et al.* 2008; Chang *et al.* 2010) or by otherwise dispensable domains of the essential branchpoint binding protein Msl5 (Chang *et al.* 2012). Nonetheless, there are two situations in which the lack of TMG caps *per se* elicits a profound phenotype. First, *S. cerevisiae tgs1*∆ diploids are unable to properly execute meiosis and sporulation because they are defective in splicing certain meiotic pre-mRNAs (Qiu *et al.* 2011). Second, although *S. cerevisiae* haploid *tgs1*∆ cells grow normally at 30°–37°, they are unable to grow at 18°–20° (Mouaikel *et al.* 2002; Hausmann *et al.* 2008), signifying that one or more essential cellular transactions becomes reliant on TMG caps at low temperatures.

To gain insights to the basis for *tgs1*∆ cold sensitivity, we sought to identify genetic suppressors that restore the growth of *tgs1*∆ cells at restrictive temperature . Based on our findings that the residual m^7^G cap of the U1 snRNP in *tgs1*∆ cells is accessible to and occupied by nuclear CBC, we queried whether mutating the cap-binding site of CBC (in the Cbc2 subunit of yeast CBC) might suppress *tgs1*∆ *cs* growth. We thereby identified a series of hypomorphic mutations of Cbc2 predicted to weaken cap-binding (Mazza *et al.* 2002; Calero *et al.* 2002), which had no effect on vegetative growth in *TGS1* cells yet restored the growth of *tgs1*∆ cells at 18°–20° (Schwer *et al.* 2011; Qiu *et al.* 2012) . We inferred from these results that the *cs* phenotype is caused, at least in part, by the ectopic association of nuclear CBP with the m^7^G cap of U1 snRNA.

This focused our attention on the U1 snRNP as a potential source of additional *tgs1*∆ suppressors. Yeast U1 snRNP consists of a 568-nt U1 snRNA, a 7-subunit Sm protein ring, and 10 U1-specific snRNP subunits: Prp39, Prp40, Snu71, Snu56, Luc7, Prp42, Nam8, Snp1, Mud1, and Yhc1 (Gottschalk *et al.* 1998; Schwer *et al.* 2011). The Nam8 and Mud1 subunits are inessential for vegetative growth. Rather than suppressing *tgs1*∆, we learned early on that *nam8*∆ and *mud1*∆ deletions were synthetic lethal with *tgs1*∆ (Hausmann *et al.* 2008). The essential Yhc1 subunit (the yeast homolog of human U1-C) interacts directly with the conserved U1 snRNA 5′ leader sequence m^2,2,7^GpppAUACUUACCU that base-pairs to the complementary sequence of the consensus yeast 5′ splice site (GUAUGU) (Kondo *et al.* 2015). Extensive mutagenesis of Yhc1, by C-terminal truncation and structure-guided alanine scanning, has yielded a collection of hypomorphic *YHC1* alleles that have no effect *per se* on vegetative growth, but have strong negative genetic interactions with Mud1, Nam8, and Mud2 (Schwer and Shuman 2014, 2015). Testing the *YHC1* mutant collection for interactions with *tgs1*∆ uncovered many instances of synthetic lethality and sickness, but no case in which the *tgs1*∆ *cs* phenotype was suppressed. The same was true of a series of truncation and alanine mutants of the SmD3 and SmB subunits of the yeast Sm ring (Schwer and Shuman 2015).

Despite these discouraging results, we continued the search for *tgs1*∆ suppressors along two lines, one U1-centric, and one unbiased. As we report here, both approaches bore fruit. In the first instance, we generated a series of viable truncation mutants of U1 subunit Snp1 and screened them for genetic interactions. We found two C-terminal truncations of Snp1 that restored the growth of *tgs1*∆ cell at low temperature.

In parallel, we screened a 2-µ plasmid-based yeast genomic library for candidate dosage suppressors of the *tgs1*∆ *cs* defect. The rationale was that bypass by overexpression might identify specific gene products or cellular transactions that are limiting when TMG caps are absent. We report the results of the screen, which identified the RNA polymerase subunit Rpo26 as capable of reviving the growth of *tgs1*∆ cells at restrictive temperature when expressed from plasmid vectors. Rpo26, a 155-aa polypeptide, is an essential constituent of all three nuclear RNA polymerases (Pol I, Pol II, and Pol III) (Archambault *et al.* 1990; Woychik *et al.* 1990). Rpo26 plays key roles in nuclear RNA polymerase assembly and function (Nouraini *et al.* 1996a; Tan *et al.* 2003). It makes direct atomic contacts to the catalytic Rpo21 subunit of yeast Pol II (Cramer *et al.* 2001), and hypomorphic mutations of Rpo26 are synthetic lethal in combination with an *rpo21-ts* allele (Archambault *et al.* 1990; Nouraini *et al.* 1996a). Rpo26 interacts similarly with the catalytic Rpa190 subunit in the crystal structure of yeast Pol I (Fernández-Tornero *et al.* 2013) and is presumed to do so with the catalytic Rpo31 subunit of Pol III. Our screen also identified Rpo31 as a weaker *tgs1*∆ suppressor.

We proceeded to conduct a structure-guided mutational analysis of Rpo26 and thereby delineated a minimal functional domain, Rpo26-(78-155), capable of *rpo26*∆ complementation and *tgs1*∆ *cs* suppression. We identified separation-of-function mutations within this domain that abolished *rpo26*∆ complementation without affecting *tgs1*∆ suppression.

## Materials and Methods

### Snp1 C-terminal truncations and tests of function

A 1.59-kbp DNA segment bearing the *SNP1* gene (nucleotides −400 to +1190) was amplified from *S. cerevisiae* genomic DNA by PCR using primers that introduced restriction sites for inserting the gene into the yeast expression plasmids pRS316 (*CEN URA3*) and pRS413 (*CEN HIS3*). The resulting plasmids p316-SNP1 and p413-SNP1 were constructed to introduce a 5′ *Bam*HI site and a 3′ *Spe*I site immediately flanking the open reading frame. C-terminal truncation alleles *SNP1-(1-223)*, *SNP1-(1-208)*, and *SNP1-(1-193)* were generated by PCR amplification with reverse primers that introduced a STOP codon in lieu of codons for Thr224, Ser209, or Phe194 and a flanking *Spe*I site. The mutated PCR fragments were digested and inserted into p413-SNP1 in lieu of the wild-type *SNP1* gene. The plasmid-borne genes were sequenced completely to confirm that no unwanted changes were introduced during PCR and cloning.

To assess the effects of *SNP1* mutations, we first generated a haploid *snp1*∆ [p316-SNP1] strain by sporulating and dissecting heterozygous *SNP1snp1∆*::*kanMX* diploids (Open Biosystems) that had been transfected with p316-SNP1. *snp1*∆ [p316-SNP1] cells were resistant to G418 and unable to grow on medium containing 5-fluoroorotic acid (FOA). To assess the function of *SNP1* alleles, *snp1*∆ [p316-SNP1] cells were transfected with p413-SNP1 (*HIS3 CEN*) plasmids. His^+^ transformants were selected and streaked on agar medium containing FOA. The plates were incubated at 20°, 30°, and 37°, and mutants that failed to form colonies at any temperature after 8 d were deemed lethal. Individual FOA-resistant colonies of viable *SNP1* alleles were grown to mid-log phase in YPD broth and adjusted to *A*_600_ of 0.1. Aliquots (3 µl) of serial 10-fold dilutions were spotted to YPD agar plates, which were then incubated at temperatures ranging from 18° to 37°. We also developed plasmid shuffle assays to test mutational effects on *SNP1* function in *tgs1*∆, *nam8*∆, *mud1*∆, *mud2*∆, and *CBC2-Y24A* cells using standard genetic manipulations of mating, sporulation, and dissection.

### Screen for dosage suppressors of *tgs1*Δ cold sensitivity

*tgs1*∆ cells were transfected with a yeast genomic DNA library in vector YEp24 (2 μ *URA3*). Approximately 41,000 Ura^+^ transformants were plated on medium lacking uracil at 18°. The 2 μ plasmid was isolated from 20 colonies that grew at 18° and then transformed into *Escherichia coli*. Plasmids were prepared from cultures of individual ampicillin-resistant transformants. The 20 candidate suppressor plasmids were re-tested by transformation into the *tgs1*∆ strain; 16 of them rescued growth of *tgs1*∆ cells at 18°. Primers flanking the cloning site were used to sequence the ends of the genomic DNA inserts in these 16 plasmids and thereby identify the genes contained in each clone. Eight of the clones contained the *TGS1* gene. Six plasmids contained a yeast genomic DNA locus, provisionally named *DTS1* (*DTS* = deletion of *TGS1* suppressor). Two plasmids contained a different yeast genomic locus, provisionally named *DTS2*.

### Yeast expression plasmids

The following DNA fragments with flanking *Bam*HI sites at both 5′ and 3′ ends were amplified by PCR using *DTS1* or *DTS2* plasmids as templates: (i) the *RPO26* ORF and intron (544 bp) plus 419 bp and 239 bp of 5′ and 3′ flanking genomic DNA; (ii) the *MLC2* ORF (492 bp) plus 443 bp and 240 bp of 5′ and 3′ flanking genomic DNA; (iii) the *PZF1* ORF (1.3 kbp) plus 455 bp and 244 bp of 5′ and 3′ flanking genomic DNA; (iv) the *SKI3* ORF (3.0 kbp) plus 442 bp and 231 bp of 5′ and 3′ flanking genomic DNA; (v) the *AZF1* ORF (2.7 kbp) plus 441 bp and 303 bp of 5′ and 3′ flanking genomic DNA; (vi) the *TRS33* ORF plus 463 bp and 276 bp of 5′ and 3′ flanking genomic DNA; and (vii) the *YOR114w* ORF plus 475 bp and 232 bp of 5′ and 3′ flanking genomic DNA. A DNA fragment containing the *RPO31* ORF (4.4 kbp) plus 426 bp of upstream (5′) and 230 bp of downstream (3′) chromosomal DNA was amplified by PCR from the *DTS2* plasmid using primers that introduced *Sal*I sites at both the 5′ and 3′ ends. The *RPO26* intron was removed cleanly from its genomic fragment via two-stage overlap extension PCR to generate the cDNA (RPO26*) with its genomic DNA flanks and *Bam*HI terminal restriction sites. The PCR products were then restricted at the terminal sites and inserted into yeast expression vector YEp24 (2 μ *URA3*). The restricted fragments of *RPO26*, RPO26*, and *RPO31* were also inserted into yeast expression vector pRS415 (*CEN LEU2*). *RPO26* was inserted into *Bam*HI-cut pRS316 (*CEN URA3*) to yield pRS316-RPO26 for use in the plasmid shuffle assays described below. The plasmid inserts were sequenced completely to exclude the acquisition of unwanted changes during PCR amplification and cloning. Plasmids p360-TGS1 (*CEN URA3TGS1*) and pUN100-TGS1 (*CEN LEU2TGS1*) used as positive controls were described previously (Hausmann *et al.* 2008).

### Rpo26 mutants

An intron-less *RPO26* ORF was PCR-amplified with sense-strand primers designed to introduce a *Bam*HI restriction site immediately upstream of the translation start codon and an antisense primer that introduced an *Xho*I site downstream of the stop codon. The PCR product was digested with *Bam*HI and *Xho*I and then inserted into a yeast expression vector pRS425-TPI (2 μ *LEU2*) to yield pRS425-TPI-RPO26, in which expression of *RPO26* is driven by the yeast *TPI1* promoter, contained in a 2.2-kb *Pvu*II fragment from pYX132 (Novagen).

Truncated *RPO26* alleles were constructed by PCR amplification with: (i) sense strand primers that introduced a new start codon at the positions specified plus a flanking *Bam*HI site and/or (ii) antisense strand primers that introduced a new stop codon after the positions specified plus a flanking *Xho*I site. Single-alanine mutations *R79A*, *E89A*, *R97A*, *E124A*, *R135A*, *R136A*, *D145A*, and *E150A* were introduced into *RPO26-(78-155)* by two-stage PCR overlap extension with mutagenic primer oligonucleotides. The PCR products containing the mutated *RPO26* ORFs were digested with *Bam*HI and *Xho*I and inserted into *Bam*HI/*Xho*I-cut pRS425-TPI-RPO26 in lieu of the wild-type *RPO26* gene. The inserts of all plasmid clones were sequenced to exclude the acquisition of unwanted mutations during amplification and cloning.

### Plasmid shuffle assay for *rpo26*Δ complementation

A heterozygous diploid *S. cerevisiae RPO26rpo26*::*kanMX* heterozygous strain (purchased from Open Biosystems) was transformed with pRS316-RPO26. The resulting Ura^+^ diploid was sporulated and tetrads were dissected. We thereby recovered viable *rpo26*::*kanMX* haploids that were resistant to G418 and unable to grow on medium containing 0.75 mg/ml FOA (5-fluoroorotic acid), a drug that selects against the *URA3RPO26* plasmid. The *rpo26*Δ strain was used to test plasmid-borne alleles of *RPO26* for *rpo26*Δ complementation by plasmid shuffle as follows. *rpo26Δ* pRS316-RPO26 cells were transfected with pRS425-TPI-RPO26 plasmids containing wild-type or mutant *RPO26* alleles. Individual transformants were selected and patched on SD-Leu agar medium. Cells from each isolate were streaked on agar medium containing 1.0 mg/ml FOA at 30°. In cases where the *RPO26*-containing plasmid supported growth on FOA, two isolates of each mutant amplified from single FOA-resistant colonies were tested for growth by spotting 3-μl aliquots of serial 10-fold dilutions of cells (from liquid cultures grown in SD–Leu medium to mid-log phase at 30° and adjusted to *A*_600_ of 0.1) to YPD agar and incubating the plates at 18° for 7 d, 25° for 4 d, 30° for 3 d, or 37° for 2 d.

## Results

### Synthetic genetics of Snp1 truncation mutants

**Figure 1:**
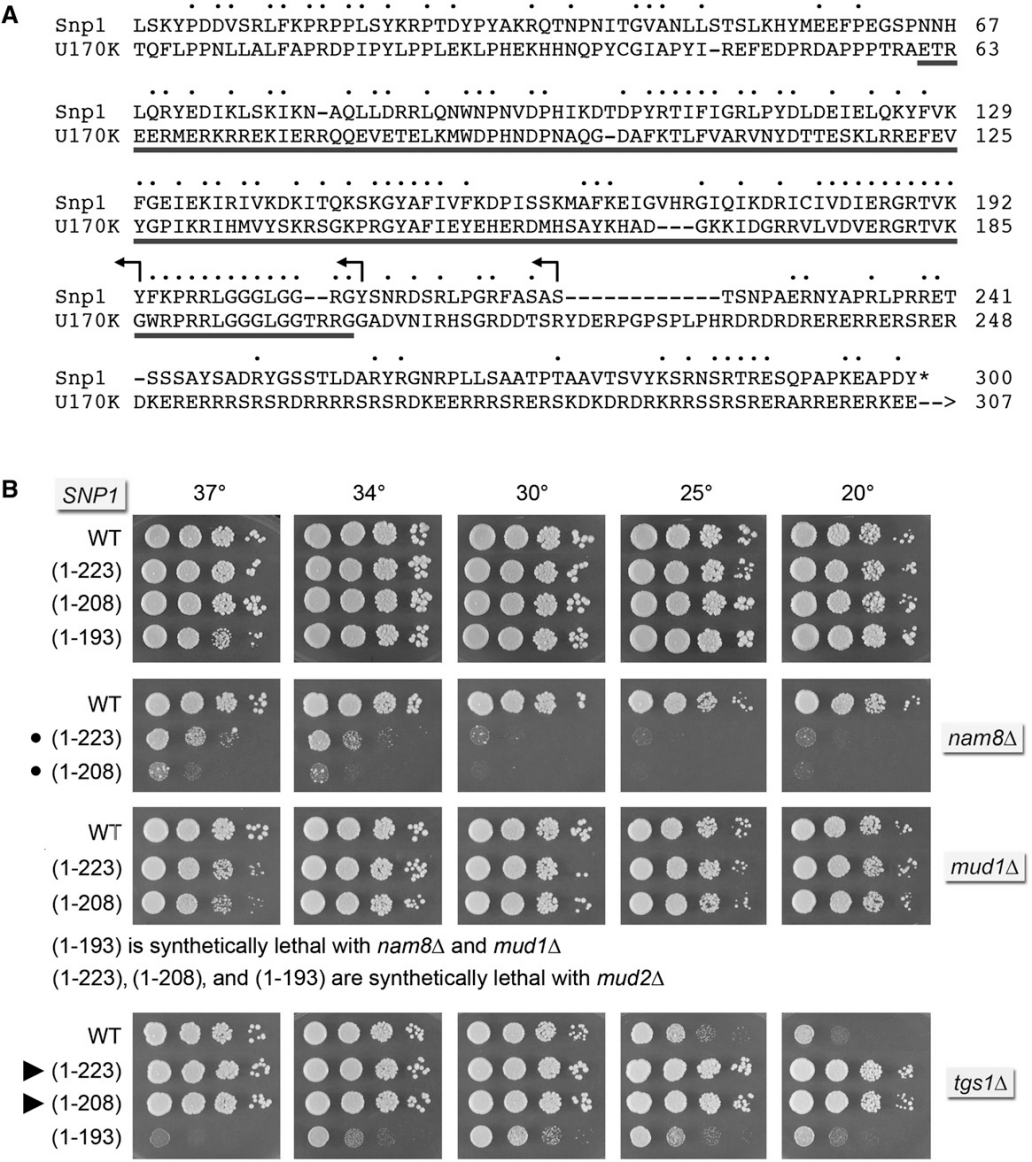
Snp1 C-terminal truncations suppress *tgs1*∆ cold sensitivity. (A) The amino acid sequence of the 300-aa *S. cerevisiae* Snp1 protein is aligned to the homologous segment of the 437-aa human U1-70K polypeptide. Positions of side chain identity/similarity are indicated by • above the alignment. Arrowheads indicate the boundaries of the C-terminal truncations of Snp1. (B) The wild-type and truncated *SNP1* alleles were tested for activity by plasmid shuffle in *snp1*∆, *snp1*∆ *mud2*∆, *snp1*∆ *nam8*∆, *snp1*∆ *mud1*∆, and *snp1*∆ *tgs1*∆ strains. Viable FOA-resistant *snp1*∆ strains bearing the indicated *SNP1* allele on a *CEN HIS3* plasmid in an otherwise wild-type (top panel), *mud1*∆, or *nam8*∆ background as indicated were spot-tested for growth on YPD agar at the temperatures specified. Synthetic growth defects are denoted by •. *snp1*∆ *tgs1*∆ strains bearing the indicated *SNP1* allele on a *CEN HIS3* plasmid were spot-tested for growth on YPD agar at the temperatures specified (bottom panel). Suppressors of the *tgs1*∆ *cs* phenotype are denoted by arrowheads.

Snp1, a 300-aa polypeptide, is the yeast homolog of human U1-70K (437-aa). Alignment of their primary structures highlights 98 positions of side-chain identity/similarity over the N-terminal 207-aa segment of Snp1 (Figure 1A). In the 3.3 Å crystal structure of the core human U1 snRNP (Kondo *et al.* 2015), the N-terminal 60-aa segment of U1-70K is a highly extended polypeptide that drapes across the surface of the U1 particle, making contacts to U1-C/Yhc1 near the U1 snRNA 5′ terminus, to each of the Sm ring subunits, and to the U1 snRNA 3′ of the Sm site. The segment of U1-70K from aa 61-202 (underlined in Figure 1A), comprising a long α helix and an RRM domain, binds to the conserved stem-loop 1 (SL1) of the U1 snRNA (Kondo *et al.* 2015). The C-terminal domains of Snp1 and U1-70K differ in length and amino acid sequence and are expected to be poorly structured based on their amino acid composition. The conserved N-terminal 21-aa peptide of Snp1 that interacts with U1-C/Yhc1 and SmD3 could be deleted without effect on yeast vegetative growth at any temperature (Schwer and Shuman 2015). A *SNP1-(22-300) tgs1*∆ double-mutant displayed the same *cs* growth defect as *tgs1*∆ (Schwer and Shuman 2015).

Here, we constructed three C-terminal truncation mutants of Snp1 with distal margins indicated by the reverse arrowheads in Figure 1A. The wild-type and truncated alleles were placed on *CEN HIS3* plasmids under the control of the native *SNP1* promoter and tested by plasmid shuffle for complementation of a *snp1*∆ p[*CEN URA3SNP1*] strain. The resulting *SNP1-(1-223)*, *SNP1-(1-208)*, and *SNP1-(1-193)* strains were viable after FOA selection and grew as well as wild-type *YHC1* cells on YPD agar (Figure 1B).

We surveyed genetic interactions of the benign Snp1 C-terminal truncations with *mud2*∆, *nam8*∆, and *mud1*∆. The results (Figure 1B) disclosed an informative hierarchy of synthetic mutational effects. *SNP1-(1-223)*, *SNP1-(1-208)*, and *SNP1-(1-193)* were lethal at all temperatures in the absence of Mud2, indicating that the essential contributions of the Snp1 segment downstream of the RRM module to early spliceosome assembly/stability are buffered by the cross-intron bridging interactions of Mud2 (engaged with Msl5 at the branchpoint) with U1 snRNP at the 5′ splice site.

*SNP1-(1-223)* and *SNP1-(1-208)* were barely viable in the *nam8*∆ genetic background and *SNP1-(1-193)* was synthetically lethal with *nam8*∆. By contrast, *SNP1-(1-223)* and *SNP1-(1-208)* supported normal growth of *mud1*∆ cells at 20°–34° and slightly slowed growth at 37° (Figure 1B). The salient finding was that the more truncated *SNP1-(1-193)* allele was synthetically lethal in the *mud1*∆ strain, signifying that the Snp1 peptide ^194^FKPRRLGGGLGGRGY^208^ is critical for U1 snRNP function *in vivo* in the absence of Mud1. The corresponding peptide in U1-70K makes direct contacts to the SL1 loop (Kondo *et al.* 2015).

### *SNP1-(1-223)* and *SNP1-(1-208)* suppress *tgs1*Δ

The standout finding was that the *SNP1-(1-223)* and *SNP1-(1-208)* truncation alleles restored the growth of *tgs1*∆ cells at 25° and 20° (Figure 1B) and at 18° (not shown). Colony size of the *SNP1-(1-223) tgs1*∆ and *SNP1-(1-208) tgs1*∆ strains on YPD medium at cold temperatures was indistinguishable from the *SNP1TGS1* wild-type strain (Figure 1B). Thus, deletion of the Snp1 segment from aa 224-300, downstream of the RRM domain, elicited a gain-of-function for the U1 snRNP that lacks a TMG cap. This positive genetic interaction was severed when the C-terminal truncation was extended into the RRM domain, *i.e.*, the *SNP1-(1-193) tgs1*∆ reverted to *cs* growth (*à la tgs1*∆) and acquired a new *ts* phenotype (Figure 1B).

### Mutational synergy of *SNP1-(1-223)* and *SNP1-(1-208)* with *CBC2-Y24A*

**Figure 2:**
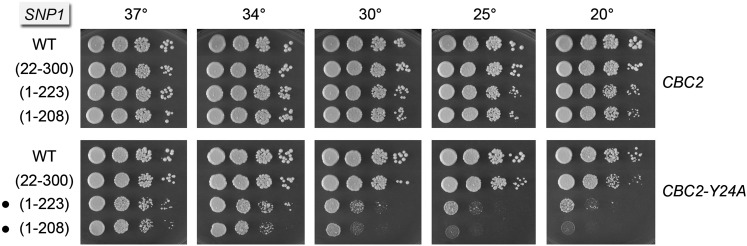
Mutational synergy of *SNP1-(1-223)* and *SNP1-(1-208)* with *CBC2-Y24A*. The wild-type and truncated *SNP1* alleles were tested for activity by plasmid shuffle in *CBC2 snp1*∆ and *CBC2-Y24A snp1*∆ strains. FOA-resistant isolates were spot-tested for growth on YPD agar at the temperatures specified. Synthetic growth defects are denoted by •.

The *Y24A* mutation in the m^7^G-binding pocket of Cbc2 suppresses the *tgs1*∆ *cs* growth defect (Qiu *et al.* 2012), as do C-terminal truncations 1-223 and 1-208 of the U1 snRNP subunit Snp1. To query potential connections between Cbc2 and Snp1, we tested by plasmid shuffle the effects of the *SNP1-(1-223)* and *SNP1-(1-208)* alleles in *CBC2snp1*∆ and *CBC2-Y24A snp1*∆ strain backgrounds. We also tested in parallel the N-terminal truncation allele *SNP1-(22-300)*, which eliminates a conserved peptide segment of Snp1/U1-70K that makes atomic contacts to the SmD3 and Yhc1/U1-C subunits of the U1 snRNP (Kondo *et al.* 2015; Schwer and Shuman 2015). Whereas *SNP1-(22-300)* caused no apparent growth defect in the *CBC2-Y24A* background, both *SNP1-(1-223)* and *SNP1-(1-208)* elicited a severe cold-sensitive defect in *CBC2-Y24A* cells (Figure 2), one that recapitulates the cold-sensitive growth defect of a *cbc2*∆ null strain (Qiu *et al.* 2012).

### *RPO26* and *RPO31* are dosage suppressors of *tgs1*Δ *cs* growth

**Figure 3:**
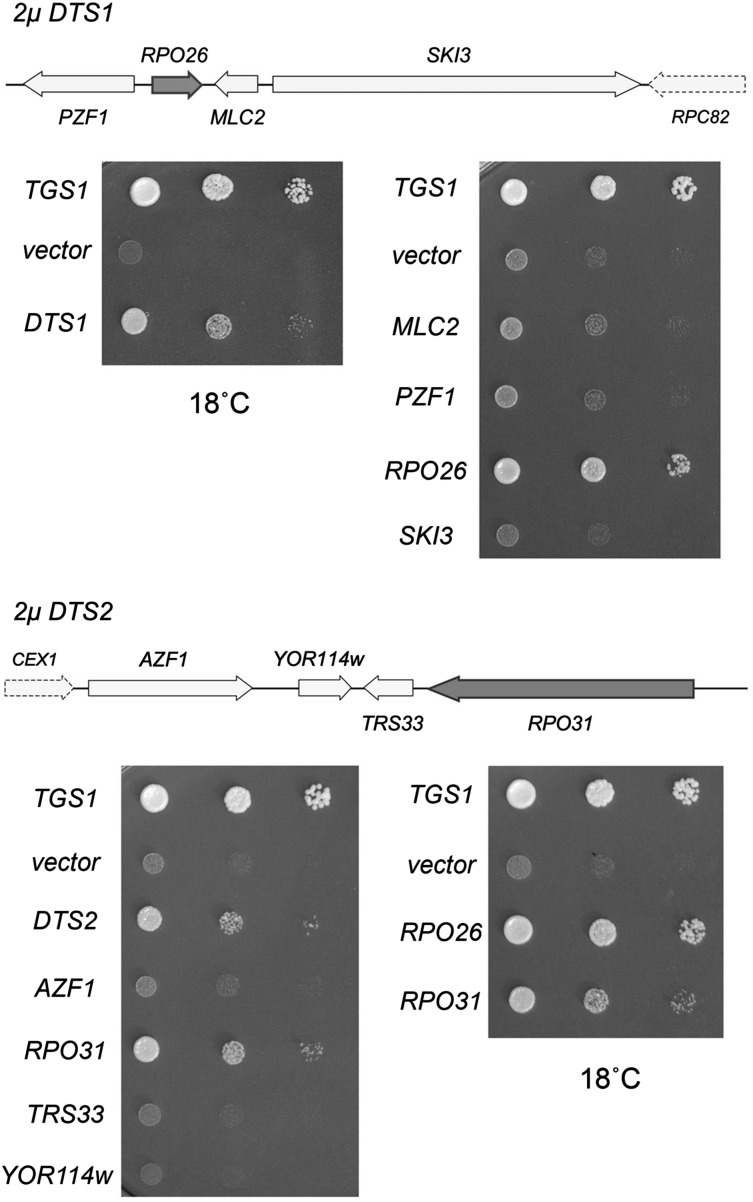
*RPO26* and *RPO31* are dosage suppressors of *tgs1Δ* cold sensitivity. *DTS1* (8.6 kb) and *DTS2* (12.4 kb) are the two genomic inserts in the 2-μ *URA3* plasmids that were isolated in the dosage suppressor screen for reversal of *tgs1*Δ *cs* growth. Individual genes with complete ORFs within the *DTS1* and *DTS2* inserts including *PZF1*, *RPO26*, *MLC2*, *SKI3*, *AZF1*, *RPO31*, *TRS33*, and *YOR114w* were cloned into a 2-μ *URA3* vector. These plasmids, an empty *CEN URA3* vector (negative control), a *CEN URA3* plasmid containing wild-type *TGS1* (positive control), and the plasmids containing *DTS1* or *DTS2* were transformed into *tgs1*Δ cells. Ura^+^ transformants were selected and grown at 30° in liquid medium lacking uracil. The cultures were adjusted to an *A*_600_ of 0.1 and aliquots of serial 10-fold dilutions were spotted on agar medium lacking uracil. The plates were photographed after incubation for 7 d at 18°.

The dosage suppressor screen entailed transformation of *S. cerevisiae tgs1*∆ cells with a 2-μ *URA3* plasmid-based wild-type genomic DNA library and selection for Ura^+^ colonies that grew at 18°. Plasmid DNA was recovered from individual yeast colonies grown at 18° and then transformed into *E. coli*. Candidate suppressors were retransformed into the original *tgs1*∆ strain and tested for growth at 18°. Sequencing the insert junctions of the plasmids that retested faithfully revealed that the rescuing clones contained either *TGS1* (as was to be expected) or one of two distinct extragenic suppressor loci, which we provisionally named *DTS1* and *DTS2*, respectively (*DTS* = deletion of *TGS1* suppressor). Note that whereas the 2-µ *DTS1* or 2-µ *DTS2* plasmids restored growth at 18°, compared to *tgs1*∆ cells carrying the empty 2-µ vector, neither 2-µ *DTS1* nor 2-µ *DTS2* was as effective as a *TGS1* plasmid, as gauged by colony size (Figure 3).

### Defining the suppressor loci within the 2µ *DTS1* and *DTS2* plasmids

*DTS1* spans a segment of chromosome XVI that includes four complete genes—*PZF1*, *RPO26*, *MLC2*, and *SKI3*—plus a 3′ fragment of the *RPC82* gene (Figure 3, top panel). To map the suppressor, we constructed a series of 2-µ vectors containing the individual *PZF1*, *RPO26*, *MLC2*, and *SKI3* open reading frames and ∼200–400 bp of 5′ and 3′ flanking genomic DNA. *tgs1*∆ cells transformed with these plasmids were tested for growth at 18°, thereby revealing that the suppressor activity was inherent to *RPO26* (Figure 3, top panel), the yeast gene encoding a shared 155-amino acid subunit of nuclear RNA polymerases I, II, and III.

*DTS2* comprises a region of chromosome XV that embraces the complete *AZF1*, *YOR114w*, *TRS33*, and *RPO31* genes, plus a 3′ fragment of the *CEX1* gene (Figure 3, bottom panel). When 2-µ vectors containing the individual *AZF1*, *YOR114w*, *TRS33*, and *RPO31* open reading frames and ∼200–400 bp of 5′ and 3′ flanking genomic DNA were transformed into *tgs1*∆ cells, only *RPO31* revived growth at 18° compared to the vector control (Figure 3, bottom panel). Rpo31 encodes the 1460-amino acid largest subunit of nuclear RNA polymerase III. Side-by-side comparison of the growth of *tgs1*∆ cells bearing 2-µ *RPO26* or *RPO31* plasmids revealed that *RPO26* was a better suppressor of the cold-sensitive defect, as gauged by colony size (Figure 3, bottom panel).

### *RPO26* and *RPO31* suppress *tgs1*Δ at low gene dosage

**Figure 4:**
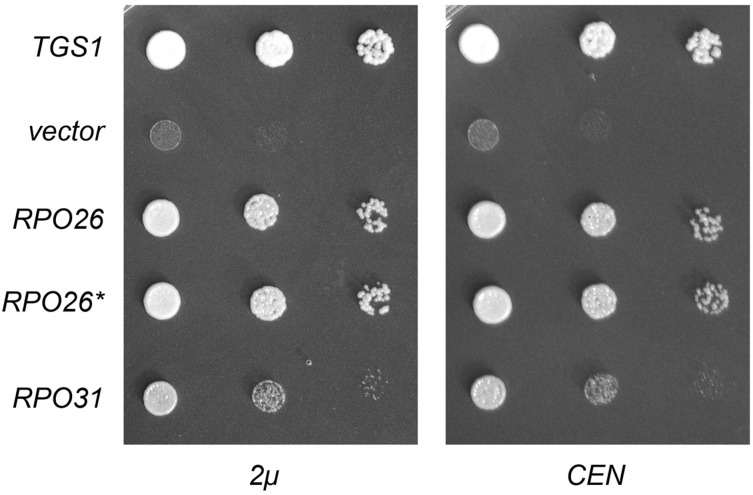
*RPO26* and *RPO31*, at low gene dosage, are capable of restoring growth of *tgs1*Δ at 18°. (Left) Yeast *tgs1*Δ cells were transformed with a *CEN URA3* plasmid bearing wild-type *TGS1* (positive control), an empty 2-μ *URA3* vector (negative control), and 2-μ *URA3* plasmids expressing wild-type *RPO26*, intron-less *RPO26* cDNA (RPO26*), or *RPO31*. Ura^+^ transformants were selected at 30° and then tested for growth at 18° by spotting serial 10-fold dilutions of liquid cultures (grown at 30° in SD–Ura medium) on Ura− agar plates. The plates were photographed after incubation for 7 d at 18°. (Right) Yeast *tgs1*Δ cells were transformed with a *CEN LEU2* plasmid bearing wild-type *TGS1* (positive control), an empty *CEN LEU2* vector (negative control), and *CEN LEU2* plasmids expressing wild-type *RPO26*, RPO26*, or *RPO31*. Leu^+^ transformants were selected at 30° and then tested for growth at 18° by spotting serial 10-fold dilutions of liquid cultures (grown at 30° in SD-Leu medium) on SD-Leu agar plates. The plates were photographed after incubation for 7 d at 18°.

The identification of two RNA polymerase subunits as dosage suppressors of *tgs1*∆ suggested a novel connection between TMG caps and transcription. The connection via Rpo31 to RNA polymerase III, which is responsible for the synthesis of many essential noncoding RNAs (5S rRNA, U6 snRNA, tRNAs), was particularly puzzling insofar as none of the known Pol III transcripts have 5′ TMG (or m^7^G) caps. One scenario that might explain the genetic suppressor results is that the loss of TMG caps affects nucleolar architecture and function (Colau *et al.* 2004) such that the assembly or activity of Pol III is compromised at cold temperature, and this defect can be overcome, in part, by overexpressing either *RPO31* or *RPO26*. If this is the case, then we might expect that simultaneously overexpressing *RPO31* and *RPO26* would afford better growth of *tgs1*∆ cells at 18° than increasing the gene dosage of either gene alone. We tested this by introducing *RPO31* and *RPO26* on the same 2-µ plasmid, but observed no better rescue of *tgs1*∆ growth in the cold than that afforded by 2-µ *RPO26* (data not shown). Another prediction of the above scenario is that *tgs1*∆ suppression should require high gene dosage. To address this issue, we placed the *RPO31* and *RPO26* genes on *CEN* plasmids and transformed them into *tgs1*∆ cells. The striking finding was that provision of *RPO26* or *RPO31* on a *CEN* plasmid was just as effective as the 2-µ *RPO26* or *RPO31* plasmids in restoring *tgs1*∆ growth at restrictive temperature (Figure 4).

The aforementioned results point toward an alternative explanation for *tgs1*∆ suppression, whereby the lack of TMG caps selectively impacts the expression of *RPO26* and/or *RPO31*, such that even one extra copy of these genes allows for growth in the cold. *RPO26* seemed to us the more plausible target of such an effect, because: (i) TMG caps are certainly implicated genetically in pre-mRNA splicing; (ii) the *RPO26* gene contains an intron, whereas *RPO31* does not; and (iii) prior studies had shown that a 60% reduction in the level of mature *RPO26* mRNA (caused by a mutation in the *RPO26* promoter) resulted in a cold-sensitive growth defect (Nouraini *et al.* 1996b). We initially considered a scenario in which adequate Rpo26 expression might somehow require the presence of an intron in the pre-mRNA, akin to what has been described for the yeast Sus1 and the intron-containing *SUS1* pre-mRNA (Cuenca-Bono *et al.* 2011; Hossain *et al.* 2011). In that case, we would expect that an intron-less cDNA version of *RPO26* would not be able to suppress *tgs1*∆. However, we found that the *RPO26* cDNA (designated RPO26* in Figure 3) was just as effective as the native *RPO26* gene in promoting *tgs1*∆ growth at 18°, whether delivered on a 2-µ vector or a *CEN* vector (Figure 4).

### N- and C-terminal truncations of Rpo26 delineate a minimal functional domain

**Figure 5:**
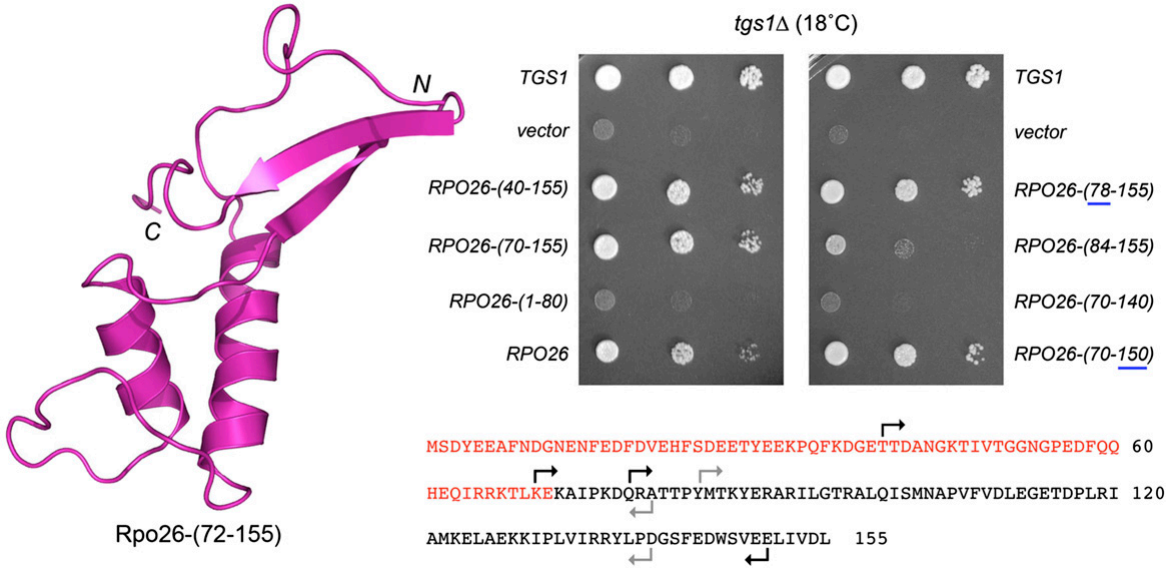
N- and C-terminal truncations of Rpo26 delineate a minimal functional domain for *tgs1*∆ suppression. (Left) Tertiary structure of Rpo26-(72-155), from the yeast Pol II crystal structure (pdb 1I3Q), with the N and C termini indicated. (Bottom right) The amino acid sequence of yeast Rpo26. The C-terminal segment visualized in the Pol II crystal structure is in black font; the disordered N-terminal segment is in red font. The margins of the N- and C-terminal truncations are denoted by forward and reverse arrows. For the N-terminal deletions, the arrows specify the residues that were mutated to methionine to initiate the truncated proteins. Black arrows denote the truncations that allow the mutants to restore *tgs1*Δ growth at 18°, whereas the gray arrows denote the truncations that disable *tgs1*∆ suppression. (Top right) *tgs1*Δ cells were transformed with a *CEN LEU2* plasmid bearing wild-type *TGS1* (positive control), an empty 2-μ *LEU2 TPI1* vector (negative control), or 2-μ *LEU2 TPI1-RPO26* plasmids bearing wild-type *RPO26* or the indicated truncation mutants. Leu^+^ transformants were selected at 30° and then tested for growth at 18° by spotting serial 10-fold dilutions of liquid cultures (grown at 30° in SD-Leu medium) on SD-Leu agar plates. The plates were photographed after incubation for 7 d at 18°.

The crystal structure of yeast RNA polymerase II (Cramer *et al.* 2001) revealed the fold of the C-terminal segment of Rpo26 from amino acids 72 to 155, which comprises two α-helices and a β-hairpin (Figure 5). The N-terminal 71-amino-acid segment was disordered in the Pol II structure. In the recent crystal structure of yeast Pol I, the N-terminal 54-amino-acid segment of Rpo26 was disordered and the segment from amino acids 55 to 71 comprised an α-helix (Fernández-Tornero *et al.* 2013). A previous study had shown that deleting 42 amino acids from the N-terminus of Rpo26 did not affect the viability of yeast cells when the truncated *RPO26-∆42* allele was driven by the strong *GAL10* promoter in galactose-containing medium; however, deletion of 84 amino acids from the Rpo26 N-terminus was lethal (Nouraini *et al.* 1996a).

**Figure 6:**
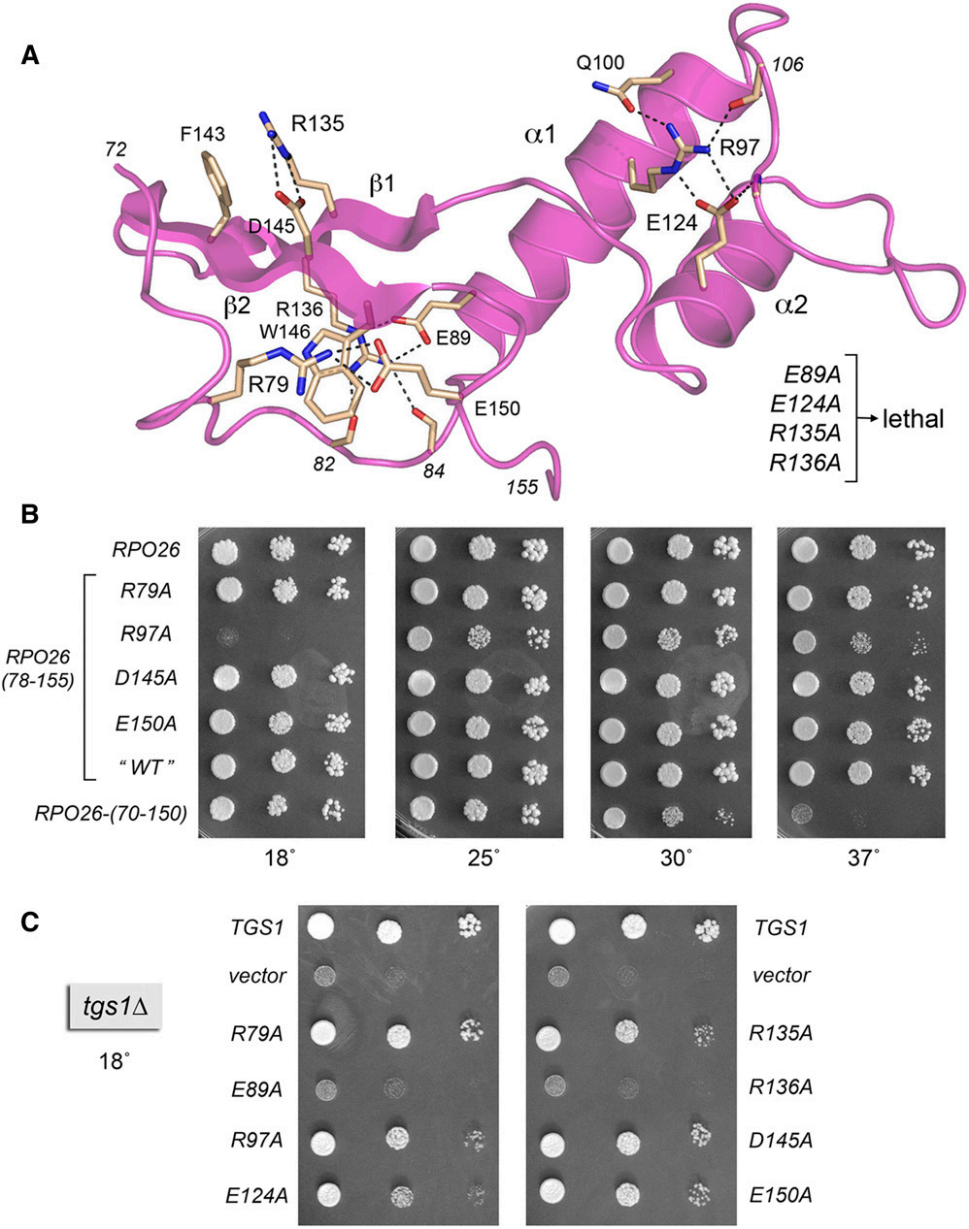
Structure-guided alanine scan of Rpo26. (A) Annotated structure of Rpo26 (from pdb 1I3Q) highlighting atomic interactions (dashed lines) of selected side chains and main chain atoms (depicted as stick models with beige carbons). Eight residues were targeted for alanine scanning: Arg79, Glu89, Arg97, Glu124, Arg135, Arg136, Asp145, and Glu150. The lethal *RPO26-Ala* alleles are indicated on the right. (B) *rpo26*∆ complementation. Yeast strain *rpo26*Δ p(*URA3 CEN RPO26*) was transformed with 2-μ *LEU2 TPI1-RPO26* plasmids bearing wild-type *RPO26*, *RPO26-(78-155)*, or the indicated *RPO26-Ala* mutants. Leu^+^ transformants were selected at 30° on agar medium containing 5-FOA (1.0 mg/ml) and aliquots of serial 10-fold dilutions of the strains with the specified genotypes were spotted on YPD agar medium. The plates were photographed after incubation for 2 d at 37°, 3 d at 30°, 5 d at 25°, or 7 d at 18°. (C) *tgs1*∆ suppression. *tgs1*Δ cells were transformed with a *CEN LEU2* plasmid bearing wild-type *TGS1* (positive control), an empty 2-μ *LEU2 TPI1* vector (negative control), or 2-μ *LEU2 TPI1-RPO26* plasmids bearing wild-type *RPO26* or the indicated *RPO26-Ala* mutants. Leu^+^ transformants were selected at 30° and then tested for growth at 18° by spotting serial 10-fold dilutions of liquid cultures (grown at 30° in SD-Leu medium) on SD-Leu agar plates. The plates were photographed after incubation for 7 d at 18°.

Here, we tested the effects of finer incremental N- and C-terminal truncations on the *in vivo* activity of Rpo26, using two genetic readouts of function: (i) dosage suppression of *tgs1*∆ and (ii) complementation of *rpo26*∆. The truncated *RPO26* alleles were placed on 2-µ plasmids under the control of the yeast *TPI1* promoter. The N-terminal deletion alleles *RPO26-(40-155)*, *RPO26-(70-155)*, and *RPO26-(78-155)* were as effective as *RPO26* in supporting *tgs1*∆ growth at 18°, whereas *RPO26-(1-80)*, a truncated version encoding just the disordered N-terminal segment of Rpo26, had no salutary effect (Figure 5). The *RPO26-(78-155)* allele complemented *rpo26*∆ in a plasmid shuffle assay. *RPO26-(78-155)* cells grew as well as wild-type *RPO26* yeast at 18°, 25°, 30°, and 37°, as gauged by colony size (Figure 6B). We conclude that the N-terminal 77 amino acids are dispensable for Rpo26 function as a subunit of the three nuclear RNA polymerases and as a suppressor of *tgs1*∆. By contrast, *RPO26-(84-155)* was a feeble suppressor of *tgs1*∆ at 18° (Figure 5) and was unable to complement *rpo26*∆ in the plasmid shuffle assay (not shown), signifying that the ^78^QRATTP^83^ peptide is important for Rpo26 activity.

We then tested the effects of deleting 5 amino acids or 16 amino acids from the C-terminus of the biologically active Rpo26-(70-155) polypeptide. Whereas *RPO26-(70-150)* was able to support growth of *tgs1*∆ cells at 18°, the *RPO26-(70-140)* allele was not (Figure 5). *RPO26-(70-140)* failed to complement *rpo26*∆ (not shown). By contrast, *RPO26-(70-150)* did complement *rpo26*∆, albeit with a conditional phenotype whereby *RPO26-(70-150)* cells grew well at 18° and 25°, formed small colonies at 30°, and failed to grow at 37° (Figure 6B). Thus, the decapeptide segment ^141^GSFEDWSVEE^150^ is necessary for Rpo21 function at warmer temperatures. Because a previous study had shown that a nonsense mutant allele encoding Rpo26-(1-145) was unable to complement *rpo26*∆ (Nouraini *et al.* 1996a), we can surmise that the pentapeptide ^146^WSVEE^150^ contains features essential for Rpo26 activity *in vivo*.

### Structure-guided alanine scan identifies amino acids essential for *rpo26*Δ complementation

The crystal structure of Rpo26 in the context of RNA polymerase II highlights a network of intramolecular side chain contacts entailing salt bridge, hydrogen bonding, and π-cation interactions (Figure 6A). Here, we performed a structure-guided alanine scan of eight residues that comprise this network: Arg79, Glu89, Arg97, Glu124, Arg135, Arg136, Asp145, and Glu150. The alanine mutations were introduced into the biologically active *RPO26-(78-155)* gene on 2-µ plasmids under the control of the *TPI1* promoter and tested for complementation of *rpo26*∆ by plasmid shuffle. Four of the alanine mutations were lethal: *E89A*, *E124A*, *R135A*, and *R136A*. Three of the alanine mutants—*R79A*, *D145A* and *E150A*—were viable and grew as well as “wild-type” *RPO26-(78-155)* at 18°, 25°, 30°, and 37° (Figure 6B). *R97A* cells grew at 25° and 30° but displayed *cs* and *ts* defects, whereby they failed to grow at 18° and grew slowly at 37°, as gauged by colony size (Figure 6B). We interpret the mutational data in light of the crystal structure, as follows.

Arg79 is located within the ^78^QRATTP^83^ hexapeptide defined as essential by our deletion analysis; Arg79 forms a salt bridge to Glu150 (Figure 6A), which is located within the essential C-terminal pentapeptide ^146^WSVEE^150^. Yet alanine mutation of either Arg79 or Glu150 was benign *in vivo* (Figure 6B), signifying that this salt bridge is dispensable and that one or more other constituents of the ^78^QRATTP^83^ and ^146^WSVEE^150^ peptides must be essential for Rpo26 function. In the case of the proximal peptide segment, we suspect that the key contributions are the hydrogen bonds of the main-chain Thr82 and Tyr84 carbonyls to the terminal guanidinium nitrogens of the essential Arg136 side chain (Figure 6A). For the distal ^146^WSVEE^150^ peptide, the Trp146 side chain is the likely key constituent, insofar as Trp146 is the focus of an extensive interaction network; it forms a cation–π–cation sandwich between Arg79 and Arg136 (Figure 6A) and it donates a hydrogen bond from Nε to the Glu144-Oε1 atom.

The essential Glu89 side chain, located in the first α-helix, forms a bidentate ion pair to the Nε and NH2 atoms of the essential Arg136 side chain, which is situated in the first β-strand (Figure 6A). Glu89 also receives a hydrogen bond to Oε1 from the main-chain amide of Thr86. We surmise that the Glu89-Arg136 salt bridge and the atomic contacts that Glu89 and Arg136 make to the main-chain of the ^82^TPYMT^86^ peptide loop preceding the first α-helix are necessary for Rpo26 folding and function. A conservative *R136K* mutation in *RPO26* elicits a temperature-sensitive growth defect (Nouraini *et al.* 1996a).

Arg135 and Asp145 are situated on the opposite face of the β-hairpin, where they form an interstrand salt bridge (Figure 6A). It was noteworthy that whereas subtracting the Asp145 side chain had no apparent impact on cell growth, the loss of Arg135 was lethal. Thus, the Asp-Arg salt bridge is not essential for Rpo26 activity. Arg135 forms a cation-π stack on Phe143 (Figure 6A), and we suspect that this cation-π interaction accounts for the essentiality of Arg135. Consistent with this idea, replacing Arg135 with lysine, which would, in principle, preserve the cation-π interaction, had no effect on yeast growth (Nouraini *et al.* 1996a).

The essential Glu124 side chain participates in a network of ionic and hydrogen bond contacts involving the two α-helices and the connecting loop (Figure 6A). Glu124 (in α1) makes a bidentate salt bridge to Arg97 (in α1 and conditionally essential at 18°) and accepts a hydrogen bond from the main-chain amide of Phe108 (in the loop). Arg97, in turn, donates hydrogen bonds to Gln100 and the main-chain carbonyl of Pro106. It was shown previously that replacing Gln110 with arginine results in cold-sensitive and temperature-sensitive growth defects (Tan *et al.* 2003).

### Rpo26 mutations that separate *rpo26*Δ complementation and *tgs1*Δ suppression activities

As one might expect, the *R79A*, *D145A*, and *E150A* mutants that complemented *rpo26*∆ at 18° were also active in suppressing *tgs1*∆ (Figure 6C). Mutations *E89A* and *R136A* that unconditionally abolished *rpo26*∆ complementation also eliminated *tgs1*∆ suppression. The salient findings were that: (i) two other mutants, *R135A* and *E124A*, that were unconditionally defective in *rpo26*∆ complementation retained *tgs1*∆ suppressor activity and (ii) mutant *R97A*, which was inactive in *rpo26*∆ complementation at 18°, nonetheless complemented *tgs1*∆ growth at 18°. Thus, *R135A*, *E124A*, and *R97A* exemplify separation of function mutations that distinguish the global role of Rpo26 in transcription by all nuclear RNA polymerases from its particular ability to act as a dosage suppressor of the cold sensitivity of *tgs1*∆ cells.

## Discussion

The present study provides new genetic insights to the impact of the lack of TMG caps in budding yeast. Prior screening for synthetic lethal and sick *tgs1*∆ interactions had drawn attention specifically to the U1 snRNP as a focal point for TMG cap function *in vivo* (Hausmann *et al.* 2008). This idea was fortified by the findings that the only overt change in the composition of yeast spliceosomal snRNPs in *tgs1*∆ cells was the gain of CBC as a stoichiometric component of the U1 snRNP, by virtue of its binding to the residual m^7^G cap on the U1 snRNA (Schwer *et al.* 2011). In *TGS1* cells, CBC is loosely associated with the U1 snRNP at low sub-stoichiometric levels compared to the intrinsic U1 snRNP subunits (Schwer *et al.* 2011). It is thought that CBC interacts with one or more of the U1 snRNP proteins to facilitate bridging interactions between CBC bound to the pre-mRNA m^7^G cap and the U1 snRNP at the 5′ splice site (Lewis *et al.* 1996; Görnemann *et al.* 2005). Hypomorphic mutations in Cbc2 that weaken cap binding suppress the *tgs1*∆ *cs* growth defect (Qiu *et al.* 2012).

Here, we show that restoration of growth of *tgs1*∆ cells in the cold can also be achieved by deleting the C-terminal 77-aa segment of the essential Snp1 subunit of the U1 snRNP. Although it had been appreciated earlier that this C-terminal portion of Snp1 was dispensable for vegetative growth (Hilleren *et al.* 1995), the genetic interactions of the Snp1-C∆ truncations were not interrogated. Underscoring the theme of redundancy in the yeast U1 snRNP, we show that otherwise benign Snp1-(1-223) and Snp1-(1-208) mutations are catastrophic in the absence of Mud2 or Nam8. Yet these same *SNP1* truncation alleles elicit a gain-of-function in the *tgs1*∆ genetic background. Our frugal speculation is that *tgs1*∆ suppression by *SNP1-C*∆ is mediated via an effect on CBC association with the residual U1 snRNA m^7^G cap, whereby the C-terminal segment of Snp1 is itself a point of contact of CBC with the U1 snRNP. In this scenario, weakening of the CBC•U1 snRNP interaction by Snp1 truncation would diminish CBC association with the U1 m^7^G cap in *tgs1*∆ cells and allow growth in the cold (more or less mimicking the Cbc2 cap-binding site mutants with respect to *tgs1*∆ suppression). In *TGS1* cells that have TMG caps, the Cbc2 cap-binding site lesion Y24A and the Snp1-C∆ truncations, which cause no growth defects *per se*, synergized when combined to mimic the severe cold sensitivity of the *cbc2*∆ null mutant. These findings are consistent with the idea that the Snp1 C-terminus contributes to the interaction of CBC with the U1 snRNP.

The C-terminal 77-aa segment of yeast Snp1 is rich in arginine (n = 11), serine (n = 12), and alanine (n = 11) and is predicted to be strongly hydrophilic, with the exception of one hydrophobic tract (^265^PLLSAATPTAAVTSVY^280^). The amino acid sequence and composition are suggestive of structural disorder or a structure that is templated by the association of this polypeptide with other proteins. Because this segment is not conserved in human U1-70K and the C-terminus of U1-70K is not seen in U1 snRNP crystal structures, we cannot intuit what contacts might be made by the Snp1 C-terminus. This will be an interesting topic for future studies given the broad impact, both positive and negative, of Snp1 C-terminal deletion on yeast physiology when other components of the splicing apparatus are simultaneously perturbed.

In a separate approach entailing a genomic library screen, we identified *RPO26* as a dosage suppressor of the cold-sensitive phenotype of *tgs1*∆ cells. Because even a nominally single extra copy of the *RPO26* gene on a *CEN* plasmid revived *tgs1*∆ growth at 18°, we surmise that *RPO26* is an especially vulnerable target of the effect of the *tgs1*∆ mutation. This vulnerability is unlikely to reflect a fastidious gene-specific requirement for TMG caps or other splicing factors in removing the *RPO26* intron, insofar as the *RPO26* 5′-splice site, branchpoint, and 3′-splice site adhere perfectly to the yeast consensus sequences and the intron is situated close to the 5′ end of the *RPO26* ORF, as is the case for most yeast genes. Rather, it is the fact that even small changes in *RPO26* expression can result in an overt growth defect in the cold (Nouraini *et al.* 1996b) that allowed us to recover a singularly sensitive target gene in the suppressor screen.

As discussed above, we implicate ectopic binding of nuclear CBC to the m^7^G cap of the U1 snRNP of *tgs1*∆ cells as a principal factor in the cold sensitivity of *tgs1*∆ cells. Mutations in the cap-binding site of CBC or deletion of the Snp1 C-terminus completely restore normal growth of *tgs1*∆ cells at 18°, unlike the dosage suppression by *RPO26*, which promotes growth of *tgs1*∆ cells at 18°, albeit not as well as *TGS1* or the hypomorphic *CBC2* and *SNP1-C∆* mutations. These findings fortify the inference that the cold sensitivity of *tgs1*∆ arises not from the lack of TMG caps, but from the effect of U1-bound CBC on vulnerable yeast mRNAs, among which *RPO26* stands out.

Our studies shed new light on the structure-activity relations of Rpo26. We refine the margins of the minimal functional Rpo26 domain, identify essential side chains by alanine scanning, and interpret the mutational effects by reference to the crystal structure of Rpo26 in RNA polymerase II. Especially instructive were the mutations that separated the *tgs1*∆ dosage suppression activity of Rpo26 from its globally essential function in nuclear transcription. An appealing explanation for this separation is that certain Rpo26 mutations selectively impact Rpo26 function in one (or two) of the nuclear RNA polymerases while sparing its function in the other polymerase(s) (Tan *et al.* 2003). In that case, we infer that the *RPO26 R135A*, *E124A*, and *R97A* mutants, which are lethal or conditionally lethal with respect to *rpo26*∆ complementation, can provide Rpo26 function for the nuclear RNA polymerase that is most affected in *tgs1*∆ cells at low temperatures. That we isolated *RPO31*, the gene encoding the largest subunit of Pol III, in the same suppressor screen that yielded *RPO26* suggests to us that Pol III is especially sensitive to the level of Rpo26 subunit in *tgs1*∆ cells at low temperatures. Rpo26 is in intimate contact with the large subunits of nuclear RNA polymerases during and after assembly of the polymerases (Wild and Cramer 2012) and there are well-documented genetic interactions of Rpo26 with Rpb1, the largest Pol II subunit (Archambault *et al.* 1990; Nouraini *et al.* 1996a). We speculate that the relatively weaker suppression of *tgs1*∆ by increased *RPO31* gene dosage (compared to *RPO26* suppression) reflects enhanced assembly of Pol III when Rpo26 levels are limiting.

## References

[bib1] Archambault J. Schappert K. T. Friesen J. T., 1990 A suppressor of an RNA polymerase II mutations of Saccharomyces cerevisiae encodes a subunit common to RNA polymerases I, II and III. Mol. Cell. Biol. 10: 6123–6131. 2247052 10.1128/mcb.10.12.6123-6131.1990 PMC362887

[bib2] Calero G. Wilson K. F. Ly T. Rios-Steiner J. L. Clardy J. C., 2002 Structural basis of m^7^GpppG binding to the nuclear cap-binding protein complex. Nat. Struct. Biol. 9: 912–917. 12434151 10.1038/nsb874

[bib3] Chang J. Schwer B. Shuman S., 2010 Mutational analyses of trimethylguanosine synthase (Tgs1) and Mud2: proteins implicated in pre-mRNA splicing. RNA 16: 1018–1031. 20360394 10.1261/rna.2082610 PMC2856874

[bib4] Chang J. Schwer B. Shuman S., 2012 Structure-function analysis and genetic interactions of the yeast branchpoint binding protein Msl5. Nucleic Acids Res. 40: 4539–4552. 22287628 10.1093/nar/gks049 PMC3378887

[bib5] Colau G. Thiry M. Leduc V. Bordonné R. Lafontaine D. L. J., 2004 The small nucle(ol)ar RNA cap trimethyltransferase is required for ribosome synthesis and intact nuclear morphology. Mol. Cell. Biol. 24: 7976–7986. 15340060 10.1128/MCB.24.18.7976-7986.2004 PMC515057

[bib6] Cramer P. Bushnell D. A. Kornberg R. D., 2001 Structural basis of transcription: RNA polymerase II at 2.8 angstrom resolution. Science 292: 1863–1876. 11313498 10.1126/science.1059493

[bib7] Cuenca-Bono B. García-Molinero V. Pascual-García P. Dopazo H. Llopis A., 2011 SUS1 introns are required for efficient mRNA nuclear export in yeast. Nucleic Acids Res. 39: 8599–8611. 21749979 10.1093/nar/gkr496 PMC3201862

[bib8] Fernández-Tornero C. Moreno-Morcillo M. Rashid U. J. Taylor N. M. I. Ruiz F. M., 2013 Crystal structure of the 14-subunit RNA polymerase I. Nature 502: 644–649. 24153184 10.1038/nature12636

[bib9] Franke J. Gehlen J. Ehrenhofer-Murray A. E., 2008 Hypermethylation of yeast telomerase RNA by the snRNA and snoRNA methyltransferase Tgs1. J. Cell Sci. 121: 3553–3560. 18840651 10.1242/jcs.033308

[bib10] Gallardo F. Olivier C. Dandjinoud A. T. Welinger R. J. Chartrand P., 2008 *TLC1* RNA nucleo-cytoplasmic trafficking links telomerase biogenesis to its recruitment to telomeres. EMBO J. 27: 748–757. 18273059 10.1038/emboj.2008.21 PMC2265757

[bib11] Görnemann J. Kotovic K. M. Hujer K. Neugebauer K. M., 2005 Cotranscriptional spliceosome assembly occurs in a stepwise fashion and requires the cap binding complex. Mol. Cell 19: 53–63. 15989964 10.1016/j.molcel.2005.05.007

[bib12] Gottschalk A. Tang J. Puig O. Salgado J. Neubauer G., 1998 A comprehensive biochemical and genetic analysis of the yeast U1 snRNP reveals five novel proteins. RNA 4: 374–393. 9630245 PMC1369625

[bib13] Hausmann S. Shuman S., 2005 Specificity and mechanism of RNA cap guanine-N2 methyltransferase (Tgs1). J. Biol. Chem. 280: 4021–4024. 15590684 10.1074/jbc.C400554200

[bib14] Hausmann S. Ramirez A. Schneider S. Schwer B. Shuman S., 2007 Biochemical and genetic analysis of RNA cap guanine-N2 methyltransferases from *Giardia lamblia* and *Schizosaccharomyces pombe*. Nucleic Acids Res. 35: 1411–1420. 17284461 10.1093/nar/gkl1150 PMC1865056

[bib15] Hausmann S. Zheng S. Costanzo M. Brost R. L. Garcin D., 2008 Genetic and biochemical analysis of yeast and human cap trimethylguanosine synthase: functional overlap of TMG caps, snRNP components, pre-mRNA splicing factors, and RNA decay pathways. J. Biol. Chem. 283: 31706–31718. 18775984 10.1074/jbc.M806127200 PMC2581544

[bib16] Hilleren P. J. Kao H. Y. Siliciano P. G., 1995 The amino-terminal domain of yeast U1–70K is necessary and sufficient for function. Mol. Cell. Biol. 15: 6341–6350. 7565787 10.1128/mcb.15.11.6341 PMC230886

[bib17] Hossain M. A. Rodriguez C. M. Johnson T. L., 2011 Key features of the two-intron *Saccharomyces cerevisiae* gene *SUS1* contribute to its alternative splicing. Nucleic Acids Res. 39: 8612–8627. 21749978 10.1093/nar/gkr497 PMC3201863

[bib18] Kondo Y. Oubridge C. van Roon A. M. Nagai K., 2015 Crystal structure of human U1 snRNP, a small nuclear ribonucleoprotein particle, reveals the mechanism of 5′ splice site recognition. eLife 4: e04986. 10.7554/eLife.04986 PMC4383343 25555158

[bib19] Lewis J. D. Izaurralde E. Jarmolowsk A. McGuinan C. Mattaj I. W., 1996 A nuclear cap-binding complex facilitates association of U1 snRNP with the cap-proximal 5′ splice site. Genes Dev. 10: 1683–1698. 8682298 10.1101/gad.10.13.1683

[bib20] Mazza C. Segref A. Mattaj I. W. Cusack S., 2002 Large-scale induced fit recognition of an m^7^GpppG cap analogue by the human nuclear cap-binding complex. EMBO J. 21: 5548–5557. 12374755 10.1093/emboj/cdf538 PMC129070

[bib21] Mouaikel J. Verheggen C. Bertrand E. Tazi J. Bordonné R., 2002 Hypermethylation of the cap structure of both yeast snRNAs and snoRNAs requires a conserved methyltransferase that is localized to the nucleolus. Mol. Cell 9: 891–901. 11983179 10.1016/s1097-2765(02)00484-7

[bib22] Nouraini S. Archambault J. Friesen J. D., 1996a Rpo26p, a subunit common to yeast RNA polymerases, is essential for the assembly of RNA polymerases I and II and for the stability of the largest subunits of these enzymes. Mol. Cell. Biol. 16: 5985–5996. 8887628 10.1128/mcb.16.11.5985 PMC231601

[bib23] Nouraini S. Hu J. McBroom L. D. B. Friesen J. D., 1996b Mutations in an Abf1p binding site in the proximal promoter of yeast *RPO26* shift the transcription start sites and reduced the level of *RPO26* mRNA. Yeast 12: 1339–1350. 8923739 10.1002/(SICI)1097-0061(199610)12:13%3C1339::AID-YEA31%3E3.0.CO;2-C

[bib24] Qiu Z. R. Shuman S. Schwer B., 2011 An essential role for trimethylguanosine RNA caps in *Saccharomyces cerevisiae* meiosis and their requirement for splicing of *SAE3* and *PCH2* meiotic pre-mRNAs. Nucleic Acids Res. 39: 5633–5646. 21398639 10.1093/nar/gkr083 PMC3141232

[bib25] Qiu Z. R. Chico L. Chang J. Shuman S. Schwer B., 2012 Genetic interactions of hypomorphic mutations in the m^7^G cap binding pocket of yeast nuclear cap binding complex: an essential role for Cbc2 in meiosis via splicing of *MER3* pre-mRNA. RNA 18: 1996–2011. 23002122 10.1261/rna.033746.112 PMC3479390

[bib26] Schwer B. Erdjument-Bromage H. Shuman S., 2011 Composition of yeast snRNPs and snoRNPs in the absence of trimethylguanosine caps reveals nuclear cap binding protein as a gained U1 component implicated in the cold-sensitivity of *tgs1*∆ cells. Nucleic Acids Res. 39: 6715–6728. 21558325 10.1093/nar/gkr279 PMC3159458

[bib27] Schwer B. Shuman S., 2014 Structure-function analysis of the Yhc1 subunit of yeast U1 snRNP and genetic interactions of Yhc1 with Mud2, Nam8, Mud1, Tgs1, U1 snRNA, SmD3 and Prp28. Nucleic Acids Res. 42: 4697–4711. 24497193 10.1093/nar/gku097 PMC3985668

[bib28] Schwer B. Shuman S., 2015 Structure-function analysis and genetic interactions of the Yhc1, SmD3, SmB, and Snp1 subunits of yeast U1 snRNP and genetic interactions of SmD3 with U2 snRNP subunit Lea1. RNA (in press). 10.1261/rna.050583.115 PMC4436669 25897024

[bib29] Simoes-Barbosa A. Chakrabarti K. Pearson M. Benarroch D. Shuman S., 2012 Box H/ACA snoRNAs are preferred substrates for the trimethylguanosine synthase in the divergent unicellular eukaryote *Trichomonas vaginalis*. RNA 18: 1656–1665. 22847815 10.1261/rna.034249.112 PMC3425780

[bib30] Tan Q. Prysak M. H. Woychik N. A., 2003 Loss of the Rpb4/Rpb7 subcomplex in a mutant form of the Rpb6 subunit shared by RNA polymerases I, II, and III. Mol. Cell. Biol. 23: 3329–3338. 12697831 10.1128/MCB.23.9.3329-3338.2003 PMC153193

[bib31] Wild T. Cramer P., 2012 Biogenesis of multisubunit RNA polymerases. Trends Biochem. Sci. 37: 99–105. 22260999 10.1016/j.tibs.2011.12.001

[bib32] Wilmes G. M. Bergkessel M. Bandyopadhyay S. Shales M. Braberg H., 2008 A genetic interaction map of RNA-processing factors reveals links between Sem1/Dss1-containing complexes and mRNA export and splicing. Mol. Cell 32: 735–746. 19061648 10.1016/j.molcel.2008.11.012 PMC2644724

[bib33] Woychik N. A. Liao S. M. Kolodziej P. A. Young R. A., 1990 Subunits shared by eukaryotic nuclear RNA polymerases. Genes Dev. 4: 313–323. 2186966 10.1101/gad.4.3.313

